# Prospects for the application of infectious virus detection technology based on propidium monoazide in African swine fever management

**DOI:** 10.3389/fmicb.2022.1025758

**Published:** 2022-09-30

**Authors:** Dexin Zeng, Bingxu Qian, Yunfei Li, Kai Zong, Wanqing Peng, Kai Liao, Xiaofeng Yu, Juanjuan Sun, Xiaying Lv, Liu Ding, Manman Wang, Tingting Zhou, Yuan Jiang, Jinming Li, Feng Xue, Xiaodong Wu, Jianjun Dai

**Affiliations:** ^1^MOE Joint International Research Laboratory of Animal Health and Food Safety, Nanjing Agricultural University, Nanjing, China; ^2^Technical Center of Hefei Customs, Hefei, China; ^3^Sanya Institute of Nanjing Agricultural University, Sanya, China; ^4^Technology Center of Hefei Customs, Anhui Province Key Laboratory of Analysis and Detection for Food Safety, Hefei, China; ^5^Animal, Plant and Food Inspection Center of Nanjing Customs, Nanjing, China; ^6^China Animal Health and Epidemiology Center, Qingdao, China

**Keywords:** African swine fever, ASFV, PMA, infectious virus, non-infectious virus

## Abstract

African swine fever (ASF) is a hemorrhagic and often fatal disease occurring in domestic pigs and wild boars. ASF can potentially greatly impact the global trade of pigs and pork products and threaten global food security. Outbreaks of ASF must be notified to the World Organization for Animal Health. In this study, we analyzed the feasibility of applying propidium monoazide (PMA) pretreatment-based infectious virus detection technology to ASF prevention and control and investigated the prospects of applying this technology for epidemic monitoring, disinfection effect evaluation, and drug development. PMA as a nucleic acid dye can enter damaged cells and undergo irreversible covalent crosslinking with nucleic acid under halogen light to prevent its amplification. Although this technology has been widely used for the rapid detection of viable bacteria, its application in viruses is rare. Therefore, we analyzed the theoretical feasibility of applying this technology to the African swine fever virus (ASFV) in terms of gene and cell composition. Rapid infectious ASFV detection technology based on PMA pretreatment would greatly enhance all aspects of ASF prevention and control, such as epidemic monitoring, disinfection treatment, and drug development. The introduction of this technology will also greatly improve the ability to prevent and control ASF.

African swine fever (ASF) is a highly contagious infectious disease in animals caused by the African swine fever virus (ASFV). ASF is characterized by a wide range of clinical symptoms, from subclinical symptoms to sudden death, that often occur within 10 days, but symptoms can occur as early as 4 days after infection (Wang et al., 2020e). As the only member of the *Asfarviridae* family, the ASFV is a large, enveloped, double-stranded DNA virus that is directly and indirectly transmitted by soft ticks (Ma et al., 2020). The virus encodes 150–165 proteins with essential functions in virus replication (Sánchez-Cordón et al., 2018). Based on the conserved gene *B646L* encoding the viral protein P72, ASFV can currently be classified into 24 genotypes (Wang et al., 2020d). The 2018 outbreak of ASF in China resulted in the severe economic losses in the pig industry (Tao et al., 2020).

At present, owing to the lack of effective vaccines and specific therapeutic drugs, ASF prevention and control depends mainly on biosafety, timely detection, culling, and disinfection. In countries without ASF, biosafety and detection are the primary methods of prevention and control, whereas countries where epidemics occur rely mainly on detection, culling, and disinfection. Early virus detection requires highly sensitive and specific diagnostic methods as this enables the rapid implementation of the necessary disease control and eradication measures (Wang et al., 2020d). Because of its sensitivity and specificity, molecular biology has been widely used to detect the ASFV; however, it has certain disadvantages. Conventional molecular biological methods cannot distinguish live virus from dead virus, which can lead to nucleic acid contamination that may cause unnecessary panic and economic losses during biosafety control and routine monitoring. Moreover, uninfected animals in the epidemic area may be contaminated by nucleic acid in the environment during the sampling process, resulting in false-positive results. In addition, molecular biological detection methods cannot be used to evaluate disinfection effects. Therefore, to address these limitations, we introduce a molecular biological method for live cell detection and consider its application prospects in the prevention and control of ASF.

## African swine fever epidemic

**FIGURE 1:**
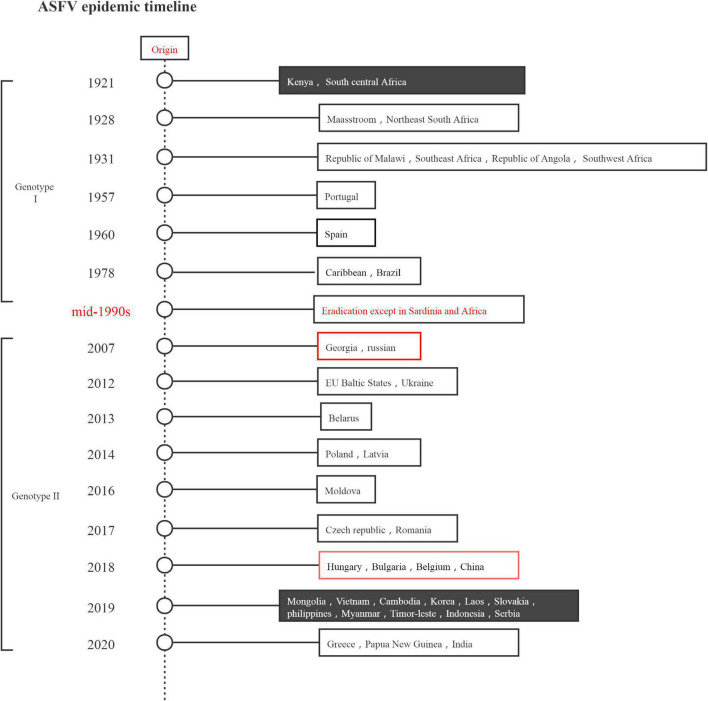
African swine fever virus epidemic timeline.

ASF was first reported in Kenya in 1921 and has subsequently spread throughout most of sub-Saharan Africa. In 1957 and 1960, it spread across the continent to Spain and Portugal, respectively, and from there to other countries in Europe, South America, and the Caribbean. The 2018 outbreak of ASF is the second transcontinental spread since 2007, with the virus spreading initially to Georgia in the Caucasus and subsequently to neighboring countries and Eastern Europe, possibly from the southeastern region of Africa. To date, ASFV has spread rapidly through China, Mongolia, Vietnam, Cambodia, Laos, the Philippines, Myanmar, South Korea, Indonesia, and other Asia-Pacific countries (Ma et al., 2020; Wang et al., 2020d; Wu et al., 2020; Yoo et al., 2020). Figure 1 presents the epidemic situation in detail. The epidemiology of the current ASF epidemic was considered to comprise three independent hints: the wild boar, the habitat, and the principal responsibility of humans in introducing the virus into domestic pig farms, which currently has high genetic and antigenic diversity. To date, 24 genotypes and 8 serogroups have been identified globally (Wang et al., 2018). For the different strain and different immunological status of the host, ASFV infection leads to a wide range of clinical presentations, varying from peracute to chronic disease, as well as an apparently asymptomatic course (Chenais et al., 2019).

## Economic impact

Among all animal diseases, ASF is one of the most important viral diseases affecting the pig industry. It has serious social and economic impacts because its detection can often lead to international trade restrictions on pork products, resulting in potentially substantial losses for the pig industry (Yoo et al., 2020; Stončiūtë et al., 2021). The difficulty in accurately estimating the overall economic costs of ASF leads to substantially varying estimates.

The disease entered Cuba in the 1980s, resulting in economic losses of $9.4 million (Negrin and Frías Lepoureau, 2002). In the last 5 years of the implementation of the eradication program alone, Spain spent an estimated $92 million (Arias et al., 2002). Based on the impact of ASF on pork production and trade and its eradication costs.

Rendleman and Spinelli (Rendleman and Spinelli, 1994) estimated the net proceeds of preventing ASF entry into the US at nearly $450 million, equivalent to 5% of total sales of US pork products. In Russia, the ASF-induced loss in 2011 was estimated at $267 million. Owing to the outbreak of ASF in Poland, Lithuania, Latvia, and Estonia in 2014 and 2015, the value of exports of pork and its products was reduced by $961 million, up to 50% of exports (Sánchez-Cordón et al., 2018). According to statistics from the national bureau of statistics of China, in 2019, the numbers of both live pigs and pork products in China decreased year-on-year. In 2019, China’s pork export volume was 210,000 tons, and a year-on-year decrease of 36.17% had with massive consequences for international trade (Wu et al., 2020). In endemic countries, the economic implications of ASF for both individual breeders, especially small-scale farmers, and national animal husbandry are considerably huge (Fasina et al., 2012); this is also true in Africa, especially for countless poor families who raise pigs for a living.

## Prevention and control of African swine fever and the associated difficulties

Although ASF was first described almost a century ago, its control has proven challenging. After the virus entered ASFV-free countries, strict quarantine with increased biosecurity, animal movement restriction, and slaughter of the affected animals remain as the only available effective control measures. No vaccines or specific medicines are available (Zakaryan and Revilla, 2016; O’Neill et al., 2020). The many epidemiologic cycles of ASF also make it a difficult disease to eradicate. Until recently, ASF epidemiology was considered to involve three independent epidemiologic cycles: sylvatic, tick–pig, and domestic, all involving soft *Ornithodoros* spp. ticks, wild African pigs (mainly warthogs), domestic pigs, and pig-derived products such as pork (Costard et al., 2013; Chenais et al., 2019). ASFV has a strong tolerance and exhibits a remarkable ability to survive for long periods in a protein-rich environment and to maintain stability at pH 4–10 (Geering et al., 2001; Bellini et al., 2016). In hams cured by salting and drying, ASFV is viable for up to 300 days. Furthermore, the virus is stable in feces at room temperature for 11 days, pig blood at 4°C for 18 months, and putrefied blood for at least 16 days (McKercher et al., 1978; Mebus, 1988; Kleiboeker, 2002). Therefore, various countries have formulated corresponding measures for the prevention and control of ASF; however, these are often difficult to implement. Small and medium-scale farms with low biosecurity levels have posed a great challenge to ASF epidemic control by creating conditions for its breeding and rapid spread and by greatly increasing the obstacles to and burdens on epidemic prevention in tracing the course of the epidemic (Wu et al., 2020). The clinical diagnosis of ASF is further complicated by the disease’s wide range of clinical forms and complex epidemiology and the similarity of its symptoms with those of other viral infections; these also increases the difficulty of ASF prevention and control (Gallardo et al., 2015; Wu et al., 2020). The lack of in-depth understanding of the prevalence, transmission mechanism, and pathogenicity of ASF has further hindered the prevention and treatment of ASF. However, it is certain that human-mediated spread of ASFV continues to play a critical role in the ASF epidemiology, despite all well-established measures currently being taken. Epidemiological studies of 68 outbreaks from August to November 2018 identified that 19% of ASF outbreaks are caused by transregional transportation of live pigs and pork products, 46% are caused by vehicles and movement of people, and 34% are caused by swill feeding (Wu et al., 2020).

## Detection methods and gaps

To date, despite the considerable efforts invested in scientific research on ASF, no safe and effective vaccine or drug has been identified (Wang et al., 2020b; Zhang et al., 2020). Prevention of ASFV spread and ASF outbreak relies critically on early and rapid detection of ASFV infection in commercial swine populations (Zhang et al., 2020, 2021). Various methods have been applied for the diagnosis of ASF, and the detection targets include pathogens, antibodies, as well as nucleic acids (Table 1). Pathogen detection methods include immunoblotting assay (Pastor et al., 1989); gold test strip assay (Sastre et al., 2016; Zhang et al., 2021); and virus isolation (VI) and detection of viral antigen by immunofluorescence assay (IFA) as recommended by OIE. Antibodies are detected mainly through enzyme-linked immunosorbent assay (ELISA) (Hutchings and Ferris, 2006). Methods for detecting the ASFV nucleic acid include polymerase chain reaction (PCR) (Agüero et al., 2003; Stear, 2005; Gallardo et al., 2015), loop-mediated isothermal amplification assay (LAMP) (James et al., 2010; Wang et al., 2020b), polymerase crosslinking spiral reaction (Fra̧czyk et al., 2016; Woźniakowski et al., 2017), cross priming amplification (CPA) (Yang et al., 2014; Fra̧czyk et al., 2016; Gao et al., 2018), recombinase polymerase amplification (RPA) (Wang et al., 2017; Miao et al., 2019; Zhang et al., 2020), real-time PCR (King et al., 2003; Zsak et al., 2005; Fernández-Pinero et al., 2013; Liu et al., 2017; Wang et al., 2020a), isothermal RPA (Miao et al., 2019), chimeric DNA/LNA-based biosensor (Biagetti et al., 2018), droplet digital PCR (ddPCR) (Wu et al., 2018), and CRISPR/Cas12a-mediated detection assay (Lu et al., 2020; Wang et al., 2020d). Although VI is the gold standard for diagnosing ASFV, it is a time-consuming and complicated procedure and is unsuitable for real-time disease monitoring (Oura et al., 2013). Detection of the ASFV antigen is suitable for large scale monitoring but is not adequately sensitive to detect early stage infection (Gallardo et al., 2019; Lu et al., 2020). In the case of low antibody titers, for antibody detection, it is important to note that current ELISA tests have a limited sensitivity, usually detecting antibodies only 12–14 d after infection (Gallardo et al., 2015). Among these assays, nucleic acid-based assay is the first choice for ASF owing to its high sensitivity and low cross-contamination rate. However, these assays cannot distinguish between living and dead viruses, which is a disadvantage in the prevention and control of ASF. For example, it is impossible to rule out false-positive interference caused by inactivated viruses, rapid evaluation of disinfection effects cannot be detected, and environmental monitoring cannot be effectively implemented. Therefore, the application of PMA pretreatment is necessary and economically effective in the prevention and control of ASFV.

**TABLE 1:** African swine fever detection methods.

Target	Detection method	Advantages	Disadvantages	Distinguish between infectious (live) and non-infectious (dead) virus?
Pathogen	Virus isolation (VI)	1. The gold standard for diagnosing ASFV. 2. Good specificity.	1. Timeliness is poor. 2. Not sensitive enough to detect early infection.	Yes
	Immunofluorescence assay (IFA)	Good specificity.		No
	Immunoblotting assay			
	Gold test strip assay			
Antibody	Enzyme-linked immunosorbent assay (ELISA)	1. Suitable for large scale monitoring. 2. Simple operation, strong specificity and high sensitivity.	Not suitable for detection of low antibody titers.	No
Nucleic acid	Polymerase chain reaction (PCR)	High sensitivity and low cross-contamination rate.	1. Complex operation. 2. Professional personnel and instruments.	No
	Loop-mediated isothermal amplification assay (LAMP)			
	Polymerase cross-linking spiral reaction			
	Cross priming amplification (CPA)			
	Recombinase polymerase amplification (RPA)			
	Quantitative real-time PCR (qRT-PCR)			
	Chimeric DNA/LNA-based biosensor			
	Droplet digital PCR (ddPCR)			
	CRISPR/Cas12a-mediated detection assay			

## Propidium monoazide principle and application

**FIGURE 2:**
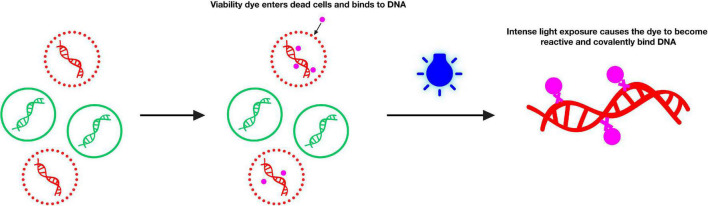
Principle of selective detection of viable cells using propidium monoazide dye (https://biotium.com/product/pmatm-dye-20mm-in-h2o/).

Currently, a viable cell detection technique based on the nucleic acid dye propidium monoazide (PMA) is widely used for pathogens (Zeng et al., 2016; Ling et al., 2020). As described previously (Zeng et al., 2016), the mechanism of the action of PMA involves the following: (i) the PMA solution selectively enters only the compromised cells when added to a mixture of intact and membrane-compromised cells. (ii) Once inside the cell, the dye intercalates into nucleic acids, whereas the azide group results in the cross-linking between the dye and DNA after exposure to strong visible light. (iii) Visible light leads to the formation of a highly reactive nitrene radical, which can react with any organic molecule in its proximity, including the bound DNA. (iv) PMA modification strongly inhibits sequential DNA amplification in PCR. (v) When the crosslinking occurs, PMA simultaneously promotes the unbound dye to react with water molecules, rendering the resulting hydroxylamine unreactive such that the DNA from cells with intact membranes is not modified in the DNA extraction (Nocker and Camper, 2009). Based on this mechanism, PMA can intercalate DNA of the dead cells, thus preventing subsequent DNA amplification of dead cells by PCR, as illustrated by Figure 2. In addition, such techniques have reportedly been used for the detection of infectious viruses related to human diseases, such as bacteriophage T4 (Fittipaldi et al., 2010), enteric viruses (Parshionikar et al., 2010; Karim et al., 2015; Leifels et al., 2015; Fongaro et al., 2016; Quijada et al., 2016), hepatitis A viruses (Sánchez et al., 2012; Leifels et al., 2015), rotaviruses (Leifels et al., 2015), adenoviruses (Leifels et al., 2015), norovirus (Karim et al., 2015), and murine norovirus (Lee et al., 2015). However, very few studies have focused on viable cell detection techniques based on PMA and molecular biology in animal disease-related viruses.

## Feasibility analysis

**FIGURE 3:**
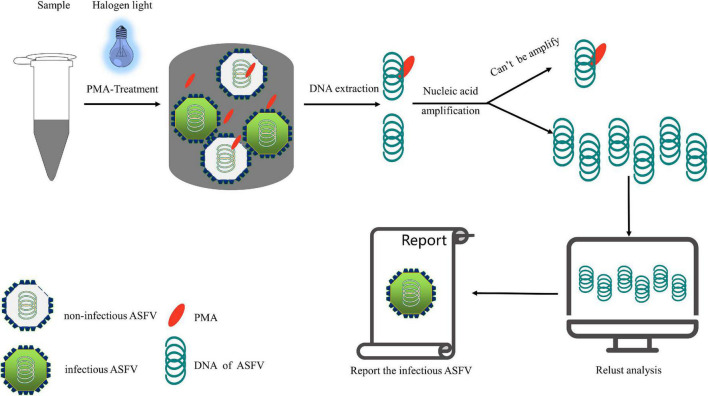
Scheme of infectious African swine fever virus detection.

From a genomic perspective, ASFV is a large double-stranded DNA virus belonging to the family Asfarviridae (Galindo and Alonso, 2017; Wang et al., 2019). Detection based on nucleic acid amplification of this relatively stable double-stranded structure does not require reverse transcription, which allows for the use of PMA-based molecular biology detection methods, similar to those used for bacteria. Architecturally, ASFV has a multilayered structure and overall icosahedral morphology. Intracellularly, it has a genome-containing nucleoid (the first layer) surrounded by a thick protein layer referred to as the core shell (the second layer), which is wrapped by an inner lipid envelope (the third layer), and an icosahedral protein capsid (the fourth layer), comprising over 50 proteins (Salas and Andrés, 2013; Wang et al., 2019). Extracellularly, the ASFV gains an external envelope (the fifth layer) as it buds through the plasma membrane (Andrés et al., 2001; Hawes et al., 2008). ASFV and gram-negative bacteria have a high degree of similarity in terms of the components of their biological barrier, which mainly comprises proteins and lipids. As PMA-based molecular biology detection technologies have been widely used for gram-negative bacteria (Li and Chen, 2012; Zhao et al., 2013; Ling et al., 2020; Zeng et al., 2020), we infer that this technology is feasible for detecting viable cells infected with ASFV. Moreover, as noted earlier, PMA-based viable cell detection technology has been successfully applied to a variety of viruses, including DNA viruses (bacteriophage T4 and adenoviruses) and RNA viruses (enteric viruses, hepatitis A virus, rotaviruses, and norovirus). The concentration of PMA used in viruses is generally 50–150 μM, differing from that used in bacteria (8–50 μM) (Fittipaldi et al., 2010; Sánchez et al., 2012; Zeng et al., 2016). This may be related to the size of the target substance, but we believe that through PMA concentration optimization and other conditions, PMA-based molecular biological detection technology can be successfully applied for the detection of infectious ASFV. We followed the reported detection process for infectious ASFV in the sample, as shown in Figure 3.

## Demand analysis

### Application in rapid detection

ASF is a highly contagious and deadly disease occurring in domestic pigs, wild boars, and other members of the *Suidae* family (Simulundu et al., 2017). ASF typically leads to acute infection, with close to 100% individual mortality at 4–9 days after exposure (Chenais et al., 2019; O’Neill et al., 2020). Therefore, in the absence of effective vaccines and drugs, rapid, and accurate detection is extremely important. However, the inability to distinguish between infectious and non-infectious viruses has proved a major obstacle to rapid detection methods using nucleic acid amplification, rendering biosafety prevention, and control systems using these rapid detection methods as monitoring tools disadvantageous. Residual nucleic acids in the infectious and non-infectious viruses also greatly interfere with the monitoring and prevention of infectious viruses. Fortunately, the PMA-based pretreatment process can solve this problem. We consider the combination of PMA pretreatment and molecular biology detection methods as a great enhancement of the diagnostic efficiency and prevention and control capabilities of ASF. Currently, in the field of virus detection, the established rapid detection methods for infectious viruses based on PMA pretreatment are mainly PMA–PCR (Parshionikar et al., 2010; Karim et al., 2015; Fongaro et al., 2016) and PMA–qPCR (Fittipaldi et al., 2010; Sánchez et al., 2012; Quijada et al., 2016). For the prevention, control, and detection of ASFV, accuracy, convenience, low-cost, and suitability for field detection are all of great significance in addition to rapid detection. Therefore, the development of other detection methods based on PMA pretreatment, such as PMA-ddPCR, PMA-RPA, PMA-CPA, PMA-LAMP, PMA-LFD, and that of related products have great research value and application prospects.

### Application in the evaluation of disinfection effect

The evaluation of the disinfection effect, a vital part of animal disease prevention and control, is directly related to the success or failure of prevention and control. Especially when ASF breaks out, culling and disinfection are the only options; therefore, the accurate evaluation of the disinfection effect becomes even more important. Cell culture is the gold standard method for testing virus infectivity (Leifels et al., 2015), and several tests are available, including 50% hemadsorption (HAD50), western blotting, indirect IFA, and cell-ELISA. For cell culture, not all enteric viruses are easy to propagate. However, ASFV propagation cannot be conducted in any cell line using conventional cell culturing methods. ASFV can be generally cultured using porcine alveolar macrophages primary cells, which are difficult to prepare and are often unstable. In a poor culture state, the virus is not susceptible or dies soon after infection, and it is impossible to perform subsequent tests. Additionally, virus propagation in cells is time consuming, labor intensive, and expensive, rendering it an unsuitable method for evaluating the disinfection effect of ASFV. As a new infectious virus detection method, molecular biology detection technology based on PMA pretreatment has many advantages, including that is has high specificity and sensitivity, is rapid, not labor intensive, inexpensive, and is convenient to perform. It is more suitable than the cell culture method for disinfection effect evaluation test. Leifels et al. (2015) used this method to determine viral infectivity after inactivation *via* heat and UV and chlorine exposure, and determined that the results as accurate as those obtained *via* conventional cell culturing methods. Using this method to evaluate the effect of different disinfection methods on ASFV will significantly improve the evaluation efficiency, which is not only conducive to the promotion of research and development of disinfection products but also can greatly save prevention and control costs and improve prevention and control capabilities.

### Application in drug research and development

For ASF, the absence of a vaccine or a viable drug treatment makes the disease extremely harmful and difficult to eradicate. Since the outbreak of the disease, all attempts by researchers to develop effective commercial vaccines, including weakened or attenuated vaccines, subunit vaccines, and DNA vaccines (Monteagudo et al., 2017; Jancovich et al., 2018) have been unsuccessful. This is mainly because ASFV is one of the largest known DNA viruses, and most of the virus’s genetic properties and functions remain unknown; there are no cell lines are available for the cultivation of ASFV for vaccine production; and ASFV has several genotypes with different phenotypic characteristics, and the vaccines tested so far have little or no cross-protection (Wang et al., 2020c). In comparison, there are far fewer barriers to drug development. According to reports of active compounds against the ASFV (Quetglas et al., 2012; Hakobyan et al., 2016, 2019; Barrado-Gil et al., 2017), it is easier to overcome the lack of ASF drugs. However, the main method that is currently in use for drug efficacy analysis is cell culturing (Hakobyan et al., 2019), which indirectly judges the efficacy of drugs by observing cell pathological changes. This method is not only time consuming and laborious but also precludes the option of obtaining real-time data, especially in animal experiments, making it impossible to understand the survival state or change trend of the virus in real-time after medicine has been administered, which greatly restricts the development of anti-ASFV drugs. The use of infectious virus detection technology based on PMA pretreatment for drug efficacy analysis makes it possible to understand the change trend of the cell culture fluid or the virus in the animal in real-time, i.e., the proportion of infectious virus and non-infectious virus, to directly reflect the effect of drugs. Notably, this method can indicate whether a certain drug is effective and also enhance the efficacy of the drug. The proficient application of infectious virus detection technology based on PMA pretreatment to the analysis of drug efficacy is expected to result in breakthroughs in the development of anti-ASFV and other antiviral drugs.

## Discussion and conclusion

ASF is a notifiable infectious disease that has a high impact on swine health and pork industry (Halasa et al., 2016). Considering that China is the world’s largest pork producer and consumer, the world’s swine industry has been greatly impacted since ASF entered China in 2018 (Wu et al., 2020). In the absence of effective vaccines and drugs, surveillance and culling are the main measures to prevent and control of ASF. Early surveillance seems to be more effective than culling. As monitoring costs are lower than culling, and monitoring does not reduce animal production and farmer’s income, farmers willingly participate in the monitoring work. However, pig farmers have no motive to participate in the monitoring without compensation to cull infectious pigs and are likely to sell or slaughter pigs, leading to further spread of the disease.

Otherwise, commonly used detection methods, such as PCR and qPCR, which are based on the amplification of nucleic acid, cannot distinguish between infectious and non-infectious viruses because dead viruses also retain intact nucleic acids. These methods cannot accurately reflect the status of infectious ASFV in the bio-samples or environment, and would not give reliable and accurate results. Other monitoring methods that target to antigen and antibody, such as ELISA and IFA, cannot yet provide accurate monitoring reports, because the dead virus may also have some intact protein components. The traditional virus infection assay can undoubtedly give the most convincing conclusion, but its detection cycle takes a long time and heavy workload. Moreover, the detection results are greatly affected by the status of somatic cells with a poor stability of the method.

In conclusion, the live ASFV detection method based on PMA pretreatment (PMA–PCR/PMA–qPCR) may give reliable results with high sensitivity, and is rapid and convenient. As a nucleic acid dye, PMA can enter the dead virus and bind nucleic acid under halogen lamp irradiation, to inhibit the amplification of nucleic acid from dead virus, which has a good application prospect. However, the pretreatment scheme of PMA will change when the virus structure varies. Based on different viruses, even the length or structure of the target fragments, researchers can optimize the PMA concentration, exposure intensity, illumination time, and dark treatment time to obtain the optimal pretreatment conditions.

More significantly, PMA–PCR/qPCR cannot only accurately detect ASFV, but also has high application potential in other fields of ASFV control, such as disinfection effect evaluation, drug research and development. Using effective disinfectants to clean infected sites, trucks, and pollutants is an important step in preventing further spread of ASF. Accurately evaluating both the efficacy of disinfectants and the effectiveness of disinfection is a key step in epidemic prevention and control and will directly affect the success of prevention and control. The efficacy of disinfectant is generally judged by the survival of the virus after disinfectant treatment. Currently, the cell culture method is commonly used. However, this method has some limitations as mentioned above, which seriously affects the efficiency of evaluating the disinfection effect. Both the complicated operation and the requirements for experimental conditions affect its practicability. In ASF epidemic areas, disinfection of pig farms is a necessary part of prevention and control. The long disinfection effect evaluation cycle often puts disinfection workers in a dilemma. Ignorance of the effect of disinfection may lead the staff to take repeated disinfection measures, which will not only result in the wastage of resources but also increase environmental pollution; moreover, incomplete disinfection, wherein the effect of disinfection is not immediately available, will increase the risk of the spread of the disease. Therefore, a method that can quickly identify infectious and non-infectious viruses is urgently needed to interpret the effect of disinfection and provide accurate information for the implementation and adjustment of ASF prevention and control programs. This rapid evaluation method should also help with the development of new disinfection products and schemes.

Drug development is also one of the ways to prevent and control ASF. Although no drugs are currently available for ASF treatment, some compounds with anti-ASFV activity have been reported. Similar to the development of disinfectants, efficacy evaluation is also critical in the development of anti-ASFV drugs. Currently, the absence of cell lines and a long evaluation cycle has hindered the development of drugs. Especially in animal experiments, a direct understanding of the change in viral trend in infected animals after drug administration will undoubtedly enhance progress in the evaluation of drug efficacy and accelerate drug screening and functional analysis. The rapid detection of infectious virus based on PMA pretreatment can be used to monitor the proportion of infectious and non-infectious viruses in animals *via* real-time PCR while reflecting the effect of drugs. Thus, the development of an infectious ASFV detection method based on PMA pretreatment will also benefit the development of anti-ASFV drugs.

In summary, rapid infectious virus detection methods are of great significance for the detection, prevention, and treatment of ASF. ASF prevention and control systems urgently need a rapid detection method based on PMA pretreatment. This technology is currently widely used for the detection of viable bacteria but rarely applied to viruses. This may be because the pretreatment of viruses by PMA is more complex, and different viruses need different treatment reminders (Leifels et al., 2015). The optimal treatment scheme of PMA for ASFV is currently unreported, presenting opportunities for research in a highly relevant field. The development and construction of molecular biological detection methods based on PMA pretreatment is urgently warranted. Rapid detection methods for infectious viruses can play a key role in all aspects of ASF prevention and control, and application prospects for effective detection methods and products hold promise.

## Author contributions

FX and DZ designed the writing idea of this manuscript. YL, KZ, XY, JS, TZ, MW, XL, and LD reviewed relevant literatures. WP and KL drew the figures. YJ, XW, JL, and JD provided technical support. DZ and BQ wrote the manuscript. FX and XW had primary responsibility for the final content. All authors have read and approved the final manuscript.
